# A Supervision Framework for Task-Shared Mental Health Workers: Implications for Clinical Trials and Beyond

**DOI:** 10.9745/GHSP-D-23-00092

**Published:** 2023-12-22

**Authors:** Stephan Rabie, Anubhuti Poudyal, Aisha King, Esona-Sethu Ndwandwa, Adele Marais, Lena Andersen, John Joska, Kathleen Sikkema

**Affiliations:** a HIV Mental Health Research Unit, Neuroscience Institute, Department of Psychiatry and Mental Health, University of Cape Town, Cape Town, South Africa.; b Department of Sociomedical Sciences, Columbia Mailman School of Public Health, New York, NY, USA.; c Department of Community Health and Health Policy, School of Public Health and Health Policy, City University of New York, New York, NY, USA.; d Department of Public Health, University of Copenhagen, Copenhagen, Denmark.

## Abstract

The rise in task-shared interventions that address the mental health treatment gap in low- and middle-income countries (LMICs) has highlighted the need for additional support and supervision of nonspecialist mental health workers (NHWs). The supervision of NHWs in most resource-limited settings is still primarily disorganized, without clear guidelines that provide the necessary structure for supervision. The need for supervision is even greater for NHWs working in the context of trauma, not only to provide training and ensure adequate delivery of care but also to provide support to minimize the psychological impact of their work. In South Africa, women face intersecting epidemics of HIV, intimate partner violence, and sexual trauma. This syndemic highlights the importance of integrating mental health treatment in HIV care, especially in settings like South Africa, where mental health services are limited. In this context, our group developed and is evaluating the effectiveness of ImpACT+, a task-shared coping intervention to improve clinical and mental health outcomes among HIV-infected women with sexual trauma in South Africa. We describe the ImpACT+ supervision model that is currently being implemented in the context of a hybrid effectiveness-implementation clinical trial. Combining experiences from clinical psychology, task-shared interventions, and trauma-informed care, the supervision model integrates formal elements of clinical supervision into categories that are suitable for use in task-shared trauma interventions in low-resource settings. To the best of our knowledge, such a trauma-informed supervision approach has not been widely documented in the literature, particularly in task-shared interventions in LMICs. In this article, we describe the ImpACT+ intervention, provide an overview of the supervision model, and provide illustrative examples of how the supervision model has been implemented.

## Abstract (precis)

The authors describe a supervision model that integrates elements of clinical supervision into categories that are suitable for use in task-shared trauma interventions in low-resource settings.

## Teaser

**Key Findings:**

- Supervision of nonspecialist mental health workers (NHWs) in most low-resource settings is still primarily unstructured, with poor evidence on the quality and quantity of supervision required to enable adequate delivery of mental health care.
- The supervision framework described in this article has 3 domains—process supervision, direct supervision, and needs-based supervision—that form a holistic approach to ensure the accurate delivery of intervention components and to support nonspecialists working in the context of trauma.
- The framework focuses on intervention fidelity and logistical support, encourages skill enhancement through ongoing appraisal of clinical skills and successful delivery of intervention components, and emphasizes the need to address experiences of vicarious trauma that pose a significant threat to NHWs working among populations with histories of trauma.

**Key Implications:**

- This framework may serve as a guide for future clinical trials of mental health interventions in low-resource settings and their eventual implementation.
- Future work should evaluate the adaptation of supervision frameworks used in clinical trials to the implementation of mental health interventions, including a detailed cost-benefit evaluation for the proposed supervision framework.

## INTRODUCTION

Globally, approximately 1 billion people are living with mental health disorders.^1^ In low- and middle-income countries (LMICs), 3 in 4 people living with mental, neurological, or substance use disorders receive no treatment in their lifetime.^1^^,^^2^ There are several reasons for this treatment gap, including structural and financial barriers, low mental health literacy, and a limited number of mental health specialists in LMICs.^3^ To address these challenges, there has been a rise in task-shared interventions that train primary care workers and nonspecialist mental health workers (NHWs) to identify and treat common mental disorders.^4^^–^^7^ In this context, we define NHWs as those who have not received specialized, in-depth professional training in mental health but are trained to diagnose and treat common mental health disorders. These interventions have been found to be effective in addressing the mental health treatment gap, especially in resource-constrained settings.^8^

Notwithstanding, the implementation of task-shared interventions has revealed some challenges. NHWs require intensive training to deliver standardized content and develop psychological micro-skills, such as verbal and nonverbal communication and empathic skills. These factors, in turn, require additional support and supervision.^7^^–^^10^ Crucially, this mental health treatment gap extends to health care providers who receive insufficient mental health treatment and support in resource-constrained settings, further compromising their ability to provide task-shared care.^11^^,^^12^ Existing supervision mechanisms for health workers in many LMICs have been critiqued as unsupportive, irregular, and demotivating.^13^^,^^14^ Some challenges that limit supervision opportunities in LMICs include a lack of mental health specialists to conduct supervision,^3^ high supervision costs,^1^ poor training opportunities for nonspecialists,^9^ inadequate supervision-focused training for specialists,^15^^,^^16^ reliance on face-to-face supervision methods resulting in the irregularity of supervision,^17^ and crucially, a lack of guidelines that provide structure to supervision.^18^ These critical supervision-related issues can impact the fidelity of experimental interventions in clinical trials and the broader adoption of mental health interventions outside the clinical trials.^17^

Supportive supervision has been broadly accepted as a model recommended for task-sharing,^19^ which includes strengthening relationships with the health system through continuous identification and resolution of problems, optimization of resources, promotion of teamwork, and open communication to improve quality delivery of services across health systems.^20^ By incorporating self- and peer-assessments, along with supervisor or expert feedback, supportive supervision moves away from the traditional supervisee-supervisor dyad into distributing the task of supervision to the entire workforce.^20^ In low-resource settings, supportive supervision has been shown to improve job performance and work satisfaction^16^^,^^21^ and is recommended for interventions to improve the performance of the health workforce.^22^ More recently, a need to focus on the internal supervision capacity and thus implement “supervisee-initiated supportive supervision” has been recommended in settings where the external capacity to conduct supervision is limited.^14^

Providing supportive supervision to NHWs is even more critical in settings with high rates of trauma, where NHWs delivering services need support not only to enhance their skills but also to manage the psychological impact of the work on themselves.^23^ Throughout trauma literature, supervision is considered a critical strategy to prevent burnout, vicarious trauma, and trauma-related stress among health care workers.^24^^–^^27^ More recently, the focus on “trauma-informed supervision” has led to approaches beyond performance enhancement.^23^^,^^24^^,^^28^ There are several characteristics of trauma-focused supervision that support NHWs working with women with trauma histories.^29^ First, trauma-informed supervision normalizes secondary traumatization as a systemic issue rather than an individual counselor's reaction, with a focus on lessening the impact of secondary traumatization. Second, rather than confrontational or punitive, trauma-informed supervision focuses on recognizing counselors' trauma reactions through adopting strategies such as peer support, reflexive training, and personal psychotherapy to support counselors.^28^ Finally, it uses strategies such as individualized coaching and performance-based feedback as a part of competence-based clinical supervision. For NHWs working in trauma-focused interventions, adopting this approach in supervision allows supervisees to gain support, share their work experiences, and advance their skills.

“Trauma-informed supervision” has led to supportive supervision approaches beyond performance enhancement that support NHWs working with clients with trauma histories.

In South Africa, women face intersecting epidemics of HIV, intimate partner violence, and sexual trauma. This syndemic highlights the importance of integrating mental health treatment in HIV care, especially in settings like South Africa, where mental health services are limited. In this context, our group developed and is evaluating the effectiveness of ImpACT+, a task-shared coping intervention to improve HIV care and mental health outcomes among HIV-infected women with sexual trauma in South Africa.^23^ The supervision model we describe is currently being implemented in the context of this hybrid effectiveness-implementation clinical trial.^30^ Combining experiences from clinical psychology, task-shared interventions, and trauma-informed care, the ImpACT+ supervision model integrates formal elements of clinical supervision into categories that are suitable for use in task-shared trauma interventions in low-resource settings. To the best of our knowledge, such a trauma-informed supervision approach has not been widely documented in the literature, particularly in task-shared interventions in LMICs. In this article, we describe the ImpACT+ intervention, provide an overview of the supervision model, and provide illustrative examples of how the supervision model has been implemented.

## THE IMPACT+ INTERVENTION

ImpACT+ is a coping intervention that seeks to improve mental health and clinical outcomes among women living with HIV with histories of sexual trauma.^30^ The intervention focuses on women initiating antiretroviral therapy, using this window of opportunity to maximize the impact on HIV care engagement. ImpACT+ is unique in several ways. Women attend 6 weekly therapy sessions that focus on exploring values that inform engaging in HIV care, recognizing the synergistic stress of sexual trauma and HIV, understanding how stressors contribute to maladaptive coping, and developing adaptive methods of coping to manage stress and improve treatment adherence. Thereafter, women attend 6 monthly maintenance sessions linked to routine clinic appointments to reinforce positive change and support the ongoing implementation of skills.

ImpACT+ goes beyond conventional cognitive-behavioral models that have traditionally been used in task-shared interventions. The intervention is directive and interactive in nature, and interventionists support women's exploration of their trauma histories and the impact on their emotions and behaviors throughout the intervention. For this reason, interventionists delivering ImpACT+ require enhanced supervision to ensure the successful delivery of the intervention content. Moreover, ImpACT+ interventionists are from similar backgrounds and communities as the women they work with, and the intervention's unique focus on sexual trauma and gender-based violence requires additional support and debriefing to mitigate the psychological effects of the interventionists' work.

Although this is a task-shared intervention, the trauma-focused nature of the intervention indicates that some prior experience in mental health is required to ensure the mental well-being of the interventionists. In ImpACT+, the interventionists are trained health care providers with a 4-year degree or diploma in nursing and/or social work, with relevant mental health training or prior experience in providing care or support to women with HIV and/or trauma histories. Nurses are the primary health care providers in South Africa, with an estimated 280,000 nurses employed in primary care in South Africa.^31^ NHWs are initially trained over a period of 5 weeks—2 weeks of intensive, in-person training, followed by 3 weeks of role-plays and content administration. NHWs are required to pass a competency assessment (a mock implementation of the ImpACT+ intervention) with a clinical supervisor before any contact with women. The NHWs are supervised by 3 postgraduate-level psychologists (master's and PhD level) who are responsible for different components of the supervision framework. Psychologists are limited in South Africa, with 0.97 psychologists per 100,000 people^8^; however, considering the trauma focus of the ImpACT+ intervention, this level of supervisor was deemed necessary.

## THE IMPACT+ SUPERVISION MODEL

Traditional models of supervision emphasize 3 main functions of the supervision process:^32^ (1) normative supervision supports logistical aspects of clinical care, including compliance with delivery protocols and ethics requirements; (2) formative supervision focuses on skill development and professional development of the care provider, including teaching additional skills in clinical practice; and (3) restorative supervision is intended to promote provider well-being and encourage job satisfaction. There has typically been an emphasis on the formative domain of supervision in both low- and high-resource settings, with less focus on the normative and restorative functions of supervision.^33^

In recognition of these limitations, we used an iterative process to develop a supervision framework for use in a Type 1 hybrid-effectiveness task-shared intervention trial.^30^^,^^34^ We built on over 2 decades of research experience implementing task-shared interventions in resource-constrained settings and conducted a targeted review of the literature on clinical supervision, task-sharing of mental health interventions in low-resource settings, and trauma-informed supervision of nonspecialists^33^^,^^35^ to inform the development of this framework. Expanding on the 3 supervision functions of the Proctor model,^32^ our framework integrates key supervision components used in ImpACT+, divided into 3 primary domains: process supervision, direct supervision, and needs-based supervision (Table). Each domain has distinct objectives but functions as a whole to ensure fidelity to key intervention components and to support nonspecialists who may experience heightened levels of distress or vicarious trauma in the delivery of an emotionally laden intervention.^36^ The 3 primary domains and their components are discussed in detail in the following sections.

The ImpACT+ supervision model functions as a whole to ensure fidelity to key intervention components and to support nonspecialists who may experience heightened levels of distress or vicarious trauma in the delivery of an emotionally laden intervention.

**TABLE.** A Detailed Overview of ImpACT+ Supervision Components

Component	Purpose	Team Member	Frequency
**Process supervision**			
Fidelity checklists	Self-reported fidelity to session content	Nonspecialist health workers	After every session
Review of session recordings	Fidelity and mastery of intervention delivery; identify areas for improvement and training	Clinical supervisor	Weekly
Quality assurance ratings	Systematic, quantitative monitoring of clinical skills	Clinical supervisor	Weekly
Supervisor memos	Monitor intervention adaptations, successes, and areas for improvement	Clinical supervisor	Monthly
Discussion with intervention specialist	Fidelity to intervention design, grasp of key concepts, and case conceptualization for difficult cases	Intervention specialist	Monthly
**Direct supervision**			
Logistic supervision meeting	Review recruitment, retention, and intervention delivery context	Project manager, senior research assistant	Weekly
Clinical supervision meeting	Session review, fidelity, and skill enhancement	Clinical supervisor	Weekly
Debriefing sessions	Containment and emotional support	Qualified mental health practitioner	Monthly
**Need-based supervision**			
Daily communication	Ongoing informal support	Whole team	As needed
Peer support	Informal containment	Nonspecialist health worker	As needed

### Process Supervision

Process supervision is a crucial step in conducting supervision in task-shared interventions, as it allows for systematic monitoring of intervention delivery and implementation while informing preparation for all other components of supervision. The main objectives of process supervision are to assess fidelity to intervention content, identify ongoing training needs, and monitor logistical challenges to intervention implementation. Process supervision is not time-bound and is completed independently by the supervisor. For example, in ImpACT+, NHWs indicate on a checklist which content was delivered during each completed intervention session. The supervisor—a clinical psychologist—independently monitors the delivery of the content by reviewing selected checklists and audio recordings of the same intervention session. Feedback on delivery and proficiency in intervention content is then provided on a weekly basis, and additional training or reinforcement is provided as required. Process supervision comprises 5 components: intervention fidelity checklists, audio recordings of intervention sessions, quality assurance ratings, supervision memos, and discussions with intervention specialists.

#### Intervention Fidelity Checklists

Fidelity checklists (Supplement 1) are the most essential component of process supervision and serve 2 main purposes. First, the fidelity checklists systematically monitor the validity and reliability of intervention delivery. Second, through monitoring intervention delivery, NHWs are continuously reminded of the intervention's key components. The checklists are completed as follows: upon delivery of each session, NHWs answer prespecified questions related to the content covered during each session, challenges experienced, and additional notes on each participant. The checklists are reviewed by the supervisor, providing additional structure to direct supervision during in-person clinical supervision meetings that occur weekly. By allowing the supervisor to identify key concerns in intervention delivery, the checklists feed into the skill development domain of supervision. In addition, checklists provide concrete, quantitative documentation of intervention sessions and fidelity, including qualitative data on sections that prove challenging to NHWs or women.

During the ImpACT+ implementation, women were provided skills to disclose their HIV status and were encouraged to do this through role-plays with the NHWs during specific sessions. However, some women found disclosure challenging and did not want to role-play during the designated sessions. As part of the process supervision step, after the session, the NHW marked the “role-play” section of the fidelity checklist as “not complete,” was prompted to explain why, and noted that the woman was not willing to participate in that part of the session. This challenge to delivery was discussed with the supervisor and collaboratively solved to address with the woman. Furthermore, these discussions with the supervisor on problem-solving could also be important for future sessions.

#### Audio Recordings of Intervention Sessions

Audio recordings of the intervention sessions are an objective way for the clinical supervisor to assess the quality of intervention delivery and NHWs' micro-skills and identify difficult points in intervention delivery. The clinical supervisor can compare NHW's perception of delivery outcomes using their completed fidelity checklist with the actual delivery using the audio recording. Audio recordings also allow NHWs to bring issues or concerns to the supervisor's attention. The supervisor listens to a random subsample (∼10%) of audio recordings for fidelity purposes, and NHWs are encouraged to suggest additional audio recordings for the supervisor to review if the NHWs have had challenging cases that require additional support and input. The audio recordings are often used to prepare discussion points and skill-based suggestions for direct supervision.

During the ImpACT+ implementation, the clinical supervisor listened to an audio recording and noticed that the NHW seemed uncomfortable when the woman discussed her sexual trauma history during the intervention session. The supervisor cross-checked the fidelity checklists and found that the NHW noted that the woman was very emotional during the intervention session. The clinical supervisor used this opportunity to help guide the NHW in ways to manage women's emotions during future sessions.

#### Quality Assurance Ratings

The clinical supervisor uses a tailored version of ENhancing Assessment of Common Therapeutic factors (ENACT)^37^ to systematically assess intervention fidelity and clinical competence of NHWs (Supplement 2). These quality assurance ratings provide additional structure to the supervision process and can optimize the time spent preparing for direct supervision. The supervisor combines quantitative ratings of performance with open-ended notes. During weekly supervision meetings, the supervisor reviews their notes with the NHWs, allowing for rich discussion. The use of quality assurance ratings depends on audio recordings of sessions or detailed session notes.

During the ImpACT+ implementation, a clinical supervisor used ENACT to assess the NHW's delivery of content, clinical skills, and ability to build rapport and make women feel comfortable during the sessions. Upon reviewing the sessions, the clinical supervisor may have noticed that the NHW did an excellent job at identifying women's need to discuss disclosure and made a note of it to reinforce this in the next clinical supervision meeting.

#### Supervision Memos

Supervision memos are monthly summaries of ongoing clinical supervision across NHWs. The clinical supervisor integrates components from audio recordings, quality assurance ratings, and synchronous clinical supervision meetings to provide succinct and structured documentation of clinical supervision. Each month, the supervisor identifies areas of concern and strength, documents alterations to intervention delivery, reflects on women's experience of the intervention, and notes her personal experience of the supervision process. The memo template (Supplement 3) provides a structured document that can be disseminated to the study team for discussions on intervention fidelity, ongoing concerns, and NHW experience of delivery.

During the ImpACT+ implementation, the clinical supervisor noted that NHWs were showing more confidence regarding guiding the women when they needed to use a coping mechanism identified as a potentially effective strategy during the intervention. The supervisor reflected on memos compiled at the start of the intervention, when the NHWs were relatively inexperienced, and noted these as areas of growth and improvement. These memos were shared with the study investigators to document progress and training needs.

#### Discussion With Intervention Specialist(s)

Building from the needs identified in supervision memos, regular meetings with local and international intervention specialists provide support for the clinical supervisor and guidance on skill development training, team management, and intervention components. Intervention implementation can be systematically discussed with both local and nonlocal experts and is integral to intervention adaptation according to local needs. Experts can provide essential information on intervention development, scientific basis, and intended implementation. Periodic discussions with experts on intervention-specific issues and modifications are also important for intervention fidelity. The time frame of these meetings will vary based on the experience levels of the NHWs and the clinical supervisor's familiarity with the intervention.

About 4 months into the ImpACT+ study, the NHWs began conducting the maintenance sessions. The clinical supervisor noted that the NHWs had been following the manual extremely closely for the maintenance sessions, which may have been leading them to ignore their clinical skills in favor of covering all the intervention content. To decide the best path forward, the clinical supervisor arranged a meeting with NHWs, the intervention developer, and the principal investigators. The nonlocal specialist discussed the importance of personally tailoring the maintenance sessions such that all potential content areas did not need to be covered and should rather be woman-centered. After this meeting, the NHWs felt more comfortable using their clinical skills to conduct the maintenance sessions.

### Direct Supervision

Direct supervision is integral for the daily functioning of the project, intervention fidelity, skill enhancement, and emotional support. The components and information gleaned from process supervision inform the content for direct supervision. Direct supervision is a synchronous, planned interaction between trained nonspecialists, supervisors, and clinical support team members. Depending on the context, direct supervision can be conducted in person or virtually. Because direct supervision is time bound, it requires a supervisor-supervisee time commitment, particularly when organizing in-person meetings. Therefore, it is more resource intensive than process supervision. Direct supervision has 3 components: logistic supervision meetings, clinical supervision meetings, and debriefing.

Direct supervision is integral for the daily functioning of the project, intervention fidelity, skill enhancement, and emotional support.

#### Logistic Supervision Meetings

Logistic supervision meetings are integral to the delivery and implementation of the intervention trial. As interventions are often delivered in real-life settings, such as primary health care clinics, hospitals, or communities, logistic supervision meetings are an opportunity for intervention members to plan the implementation of the intervention, for NHWs to receive feedback on a weekly basis on the challenges related to delivery and implementation, and to discuss potential solutions and improvements to delivery.

The ImpACT+ implementation delivery was halted for 2 weeks due to ongoing unrest and violence in the community of implementation. Women could not travel to research sites to participate in the intervention. Logistic supervision enabled the NHWs and supervisors to discuss the challenges posed by the unrest and find solutions to intervention delivery. Women completed intervention sessions by phone if they could not reschedule their intervention session until the unrest was resolved.

#### Clinical Supervision Meetings

Clinical supervision occurs weekly and aims to provide clinical support and skills training for NHWs. It is informed by process and needs-based supervision and focuses on the normative and formative supervision domains. Clinical supervision is often conducted in a group, during which supervisors and NHWs discuss individual de-identified women and themes that occur in sessions, including concerns that arose in listening to audio recordings and reading fidelity checklists. The supervisor provides refresher training on key intervention components and additional clinical training as needed. The intervention materials used by the NHWs for session delivery can also be brought to the supervision meeting to further expound on focused content and delivery of the intervention. NHWs are encouraged to note any challenges experienced in intervention delivery. For women with particularly complex treatment histories, the supervisor might go into detailed case discussions with NHWs to provide specific guidance for these women. These clinical supervision meetings are often conducted on an individual basis.

In addition to providing clinical support, these meetings also serve as a check-in of overall work progress and limitations. A virtual mechanism to conduct clinical supervision (e.g., video conferencing, text messaging, or phone calls) provides an additional way to communicate if in-person meetings are not possible. Virtual supervision is also ideal if there are limited supervisors in the area. Clinical supervision meetings can provide important insight into the debriefing needs of the NHWs. Notes from the clinical supervision meetings also inform the supervision memos.

During the ImpACT+ study, a woman presented with a severe substance use disorder, which impacted her ability to engage with the intervention content and skills. Substance misuse is not a specific focus of ImpACT+ but is among the most frequent forms of avoidant coping addressed in the intervention. The NHW brought this issue to the clinical supervision meeting, during which the need for additional training on the conceptualization of the intervention approach within the context of coping and substance misuse was identified. The supervisor used this as an opportunity for skills training so that the NHWs could learn how to approach similar situations in the future. They were then able to apply similar problem-solving and management techniques when they encountered other women who had challenges with substance use later in the intervention.

#### Debriefing

Considering ImpACT+'s unique focus on sexual trauma and abuse, the provision of debriefing and emotional support by a qualified mental health practitioner was deemed a crucial component of the supervision framework. This debriefing is distinct from single-session post-traumatic debriefing sessions, which have been shown to be ineffective among emergency responders and health care workers. NHWs empathically engage with women who have complicated histories of sexual trauma, intimate partner violence, and HIV. The monthly debriefing sessions are separate from process and direct supervision; serve to mitigate the potential for vicarious trauma, compassion fatigue, and burnout; and can assist NHWs in understanding complex emotional responses. The confidential debriefing sessions—facilitated by an independent clinical psychologist, not involved in other aspects of supervision, with experience in the fields of both trauma and HIV—create a safe space for NHWs to process how their work impacts their mental health and well-being and highlight the need for emotional processing, support, and self-care. Debriefing sessions can be conducted more frequently than every month, depending on the expressed needs of the respective NHWs. Individual debriefing sessions are made available when needed.

During the ImpACT+ implementation, an NHW shared with the group that she had started to feel emotionally and physically fatigued implementing the intervention. She struggled to “switch her mind off” from work when she got home and had started experiencing anxiety and sleep disturbances. Her family had noted that she appeared “disconnected” from them. The group had received psychoeducation about vicarious trauma, compassion fatigue, and burnout in past debriefing sessions, and the NHW noted some of the signs that resonated with her. The group shared their thoughts on how the nature of their work could be emotionally burdensome and the impact it could have on their mood and functioning. Through a facilitated conversation, the NHW reflected on some difficult emotions she felt during her engagement with specific women's stories. The similar experiences shared in the group offered validation and support, and group members discussed how various self-care practices that enhance relaxation, mindfulness, and well-being were important and helpful for them.

### Needs-Based Supervision

Needs-based supervision comprises unstructured interactions between any members of the intervention team. This domain is crucial to the daily functioning and implementation of the intervention and serves to address challenges that arise during implementation. Needs-based supervision is often spontaneous and can be in person or virtual (e.g., WhatsApp group messages throughout the workday). In this context, needs-based supervision is frequently directed by NHWs and study staff based on their needs to implement and execute the intervention. This domain has 2 components: daily communication and peer support.

Needs-based supervision—unstructured interactions between intervention team members—serves to address challenges that arise during implementation.

#### Daily Communication

To address ad hoc issues that arise during intervention delivery, NHWs need to contact supervisors and each other throughout the day for logistical support, emotional support, or questions regarding intervention delivery. The content and challenges discussed during this component also inform process supervision. Across settings, but particularly in low-resourced settings, spontaneous issues arise in the context of intervention delivery and daily operations of clinical care. Daily communications may be in person if the clinical supervisor is on-site but are frequently conducted virtually (e.g., text messages or phone calls).

During the ImpACT+ implementation, women occasionally presented with suicidality during intervention sessions. NHWs receive training in human subject protection to assess suicide risk, but trial standard operating procedures require a second clinical opinion to determine women's level of risk. Using this domain, NHWs contacted the clinical supervisor for a clinical assessment of the woman's risk status and the appropriate referral pathway scaffolded with trial-specific standardized operating procedures.

#### Peer Support

Peer support is integral for NHWs' emotional well-being. In the ImpACT+ supervision model, peers are other research staff members (i.e., research assistants and workers) working on the trial but not involved in the delivery of the ImpACT+ intervention. Considering the daily challenges associated with implementing a task-shared coping intervention focusing on sexual trauma and HIV, peer support represents an opportunity for NHWs to discuss challenging client cases with each other. Peer support offers immediate containment and emotional support for NHWs while conducting their daily activities. It can be an in-person or online platform for NHWs to talk to each other about their cases.

Both control and comparison groups in our trial^30^ include an intervention, with the control group receiving another mental health treatment (adapted problem-solving therapy).^38^ We used this treatment to provide a reasonable comparison intervention for this vulnerable client population that provided a relatively high level of care. NHWs were trained on 1 intervention. Considering these 2 groups and the potential risks for intervention contamination, NHWs were trained on which aspects of the intervention and its content could be discussed with their peers. In general, NHWs from the respective conditions are discouraged from discussing the specific content of the intervention with each other but are encouraged to offer containment and support for the emotional burden their work carries.

During the ImpACT+ implementation, an NHW felt overwhelmed after an intensely emotional intervention session, so she called another NHW to talk about the experience. The second NHW responded empathetically and provided emotional support by empathizing with her colleague and sharing how she managed similar experiences.

## DISCUSSION

The rise in task-shared interventions that address the mental health treatment gap in LMICs has highlighted the need for additional support and supervision of NHWs.^7^^–^^10^ Despite the use of various supervision models for task-shared mental health care in LMICs, supervision of NHWs in most low-resource settings is still primarily unstructured,^15^ with poor evidence on the quality and quantity of supervision required to enable adequate delivery of mental health care.^39^ For NHWs working in the context of trauma, the need for supervision is even greater—not only to provide training and ensure adequate delivery of care but also to provide support to minimize the psychological impact of their work. The lack of appropriate supervision represents a serious threat to the fidelity of experimental interventions, the well-being of NHWs, and the broader adoption of mental health interventions beyond research trials.

The supervision framework's primary domains have distinct objectives but form a holistic approach to ensure the accurate delivery of intervention components and to support nonspecialists working in the context of trauma. Our supervision framework focuses on the systematic evaluation of intervention fidelity and logistical support of the daily functioning of the intervention. Second, we encourage skill enhancement through ongoing appraisal of clinical skills and successful delivery of core intervention components. This is coupled with capacity-building and encouragement of professional development. Finally, our framework emphasizes the need for debriefing to address experiences of vicarious trauma—an understudied yet significant threat to the NHWs working among populations with a history of trauma and mental illness. Our systematic collection of multimethod supervision data feeds into generating nuanced evidence on the potential for intervention fidelity in the event of implementation at scale. For interventions to be effectively implemented at scale, we need to be able to determine the causal mechanisms of the intervention components—high levels of intervention fidelity are crucial to establish this mechanism.

The supervision framework presented in this article provides the necessary structure and supervision domains required to supervise NHWs in a resource-constrained setting. This framework is currently implemented in the context of a community-based mental health clinical trial in Cape Town, South Africa. The trial employs a variety of staff (e.g., staff, research assistants, social workers, and nurses) that require daily, weekly, and monthly supervision and support. Our supervision is conducted by 3 postgraduate-level psychologists (master's and PhD level) who are responsible for implementing different components of the framework. From an implementation perspective, this approach reduces the burden and workload on each individual supervisor while continuing to provide sufficient support for the trial staff. For this reason, the framework can be resource intensive and requires several role-players to be used at its full potential. We recognize that settings with fewer resources may not be able to employ 3 psychologists to implement this framework. However, the framework includes flexible domains that can be tailored for implementation in a resource-constrained setting outside of clinical trials. For example, fidelity checklists and supervision memos are cost-effective components that can be used to systematically monitor the delivery of interventions in settings with limited resources. These checklists and memos can be completed electronically if there is access to a computer and/or mobile phone, or alternatively, be printed and completed by hand. In settings with more resources, audio recordings of intervention sessions and supervisors who can review these recordings may be more viable. A strength of this framework is that it integrates multiple modes of supervision—WhatsApp messages, phone calls, in-person supervision, and debriefing—rather than relying only on in-person supervision alone, which has shown to be inadequate in low-resource settings.^17^ In resource-limited settings, the multiple modes of supervision presented in this framework may be beneficial for supervisors to frequently engage with NHWs, especially if supervisors are situated off-site. The frequent engagement between supervisors and NHWs, whether virtual or in person, is crucial to monitor the implementation of interventions, review progress and fidelity, and offer containment and support.

The supervision framework presented in this article provides the necessary structure and supervision domains required to supervise NHWs in a resource-constrained setting.

One of the main purposes of developing this supervision framework is to support the implementation of mental health interventions beyond the realm of clinical trials and into routine health services. There is a paucity of implementation science research focused on clinical supervision and supervision practices,^40^ particularly in low-resource settings. However, studies have documented failure to continue effective supervision when interventions move from trials to practice.^16^ There is a recent call to use supervision to increase evidence-based treatment adoption, implementation, and sustainment as mental health interventions move from research to practice.^41^ Thus, the adoption of a robust supervision model can be key to research translation because the relationship between supervision and implementation outcomes has gained increasing attention in the last decade.^41^^,^^42^ Our suggested framework—although developed within the clinical trial settings—has adaptable components that can be modified according to the program objectives, resources available, and overall human resource capacity to implement supervision as a part of national health systems' regular and ongoing mental health programs. Although this framework was designed specifically for task-shared interventions in low-resource settings, it may apply more broadly to any setting with limited mental health care professionals and bridge the gap between supervision in clinical trials and their eventual implementation and uptake in primary care settings.

In the context of a clinical trial, particularly one that evaluates the efficacy of an intervention, process supervision is essential to ensure fidelity to intervention and provide documentation to that effect. However, direct and needs-based supervision are likely to take priority in implementing and scaling up mental health interventions. Task-sharing is designed to address the issue of insufficient specialized health workers in LMICs; therefore, the need for NHWs to be adequately supervised and the demands on supervisors' time must be balanced. The requirements for supervision will vary across settings and types of mental health intervention. However, we found that emotional support and ongoing debriefing, particularly in trauma-informed work, were essential and often overlooked components of supervision for the well-being of NHWs. We encourage the inclusion of debriefing and support in all task-shared mental health interventions. In addition, we found that daily communication via digital technology, such as messaging and phone calls, is essential in this setting, both for the successful conduct of the trial and for the successful delivery of the intervention.

## CONCLUSION

We envisage that this framework will provide a guide for future clinical trials of mental health interventions in low-resource settings and their eventual implementation. Future work should evaluate the adaptation of supervision frameworks used in clinical trials to the implementation of mental health interventions, including a detailed cost-benefit evaluation for the proposed supervision framework.

## Supplementary Material

23-00092-Rabie-Supplements.pdf

## Notes

**Peer Reviewed**

**First published online:** November 29, 2023.
