# Supplementary material for: A Supervision Framework for Task-Shared Mental Health Workers: Implications for Clinical Trials and Beyond
Source: Glob Health Sci Pract. 2023 Dec 22;11(6):e2300092. doi: 10.9745/GHSP-D-23-00092 (PMC10749652; doi:10.9745/GHSP-D-23-00092)
Supplement: 23-00092-Rabie-Supplements.pdf [file 23-00092-Rabie-Supplements.pdf]

Supplement to: Rabie S, Poudyal A, King A, et al. A supervision framework for task-shared mental health workers: implications for clinical trials and beyond. *Glob Health Sci Pract*. 2023;11(6):e2300092. https://doi.org/10.9745/GHSP-D-23-00092

Supplement 1: Fidelity Checklists

Interventionist Impact Content Checklist Session 1

record id ______________________________

Session 1: Understanding the Combine Stress of HIV and Sexual Trauma

PID ______________________________

Date Delivered ______________________________

Start Time (HH:MM) ______________________________

End Time (HH:MM) ______________________________

Length ______________________________

Please indicate if the following sections were covered

Discuss participant's HIV History
- ◯ Yes
- ◯ Partly
- ◯ No

If partly or no, what was not covered and why was it not covered? ______________________________

Explore motivators for HIV care engagement: Values Exercise (island metaphor)
- ◯ Yes
- ◯ Partly
- ◯ No

If partly or no, what was not covered and why was it not covered? ______________________________

Explain intervention objectives & what to expect
- ◯ Yes
- ◯ Partly
- ◯ No

If partly or no, what was not covered and why was it not covered? ______________________________

Start strong/ stay strong visual
- ◯ Yes
- ◯ Partly
- ◯ No

If partly or no, what was not covered and why was it not covered? ______________________________

Supplement to: Rabie S, Poudyal A, King A, et al. A supervision framework for task-shared mental health workers: implications for clinical trials and beyond. *Glob Health Sci Pract*. 2023;11(6):e2300092. https://doi.org/10.9745/GHSP-D-23-00092

Supplement 2: Quality Assurance Ratings

Supervisor Impact Review Session 1

record id	________________
Interventionist Name	________________
PID	________________
Date of Session (D-M-Y)	________________
Date listened (D-M-Y)	________________
Date of supervision (D-M-Y)	________________ (Expected date of supervision, if actual supervision date is different please re-enter the form and change)

Content

Getting to Know the Participant, Relationship Building: Makes preliminary conversation to build rapport with the participant. Transitions effectively and naturally to the ImpACT model and content	◯ Needs improvement<br>◯ Done partially<br>◯ Done well<br>◯ Not applicable
Introduces goals of the intervention: Provides a preliminary explanation of the goals of the intervention, including improved coping with trauma, commitment to taking ART, and engagement with HIV care	◯ Needs improvement<br>◯ Done partially<br>◯ Done well<br>◯ Not applicable
Elicits a commitment: Obtains an agreement with the patient to participate in counselling	◯ Needs improvement<br>◯ Done partially<br>◯ Done well<br>◯ Not applicable
Start Strong, Stay Strong: Introduces the "Start Strong, Stay Strong" motto as a goal for effective care engagement	◯ Needs improvement<br>◯ Done partially<br>◯ Done well<br>◯ Not applicable
Values Bridge: Effectively introduces and completes the values bridge exercise to identify personal motivators and values related to care engagement	◯ Needs improvement<br>◯ Done partially<br>◯ Done well<br>◯ Not applicable
3H Model: Obtains brief trauma history, introduces 3H model, and assesses impact of trauma on head, heart, hands	◯ Needs improvement<br>◯ Done partially<br>◯ Done well<br>◯ Not applicable

Note: Kohrt BA, Jordans MJD, Rai S, et al. Therapist Competence in Global Mental Health: Development of the Enhancing Assessment of Common Therapeutic Factors (ENACT) Rating Scale. *Behav Res Ther*. 2015;69:11-21. doi:10.1016/j.brat.2015.03.009

Supplement to: Rabie S, Poudyal A, King A, et al. A supervision framework for task-shared mental health workers: implications for clinical trials and beyond. *Glob Health Sci Pract*. 2023;11(6):e2300092. https://doi.org/10.9745/GHSP-D-23-00092

Supplement 3: Supervision Memo Template

Month:
Year:

Introduction

Provide an overview of the past month's strengths, concerns, growth, and reflection.

Strengths

Highlight strengths identified in interventionist(s)

Areas of concern

Highlight any areas of concern regarding implementation or individual interventionist(s)

Areas for training/growth

Discuss potential areas for further growth and/or training

Reflection on participant experience of intervention

Highlight any participant experiences in the intervention.

Amendments made to the intervention this month

Suggest any amendments to the intervention.

Supervisor reflection

Highlight any reflections you may have about the progress of the intervention.
